# Inhibition of TRAF3IP2 Modulates NAMPT and NAD Metabolism in Glioblastoma

**DOI:** 10.1007/s11481-025-10252-z

**Published:** 2025-10-22

**Authors:** Kurtis Willingham, Amin Izadpanah, Rashad Yasmine, Antonia Reilich, Daneshimehr Fatemeh, Sakamuri Siva, Steven Braun, Eckhard U. Alt, Reza Izadpanah

**Affiliations:** 1 Applied Stem Cell Laboratory, Medicine/Section Cardiology, Tulane University School of Medicine, Tulane University, New Orleans, LA USA; 2 Department of Medicine, Auburn University at Montgomery, Montgomery, AL USA; 3 Department of Surgery, Tulane University School of Medicine, New Orleans, LA USA

**Keywords:** TRAF3IP2, Glioblastoma, Tumor microenvironment, Metabolism, Cell proliferation, Apoptosis

## Abstract

Glioblastoma is a grade 4 diffuse astrocytic glioma that is the most aggressive brain malignancy, with poor treatment outcomes and median overall survival (OS) of 10–14 months. Glioblastoma is characterized by upregulation of NAD metabolism, required to maintain rapid proliferation and DNA repair. Nicotinamide phosphoribosyltransferase (NAMPT), is the rate limiting enzyme in the NAD salvage pathway, and has emerged as a promising target in the treatment of glioblastoma. Previously, we reported the crucial role of adaptor protein TRAF3IP2 in glioblastoma tumorigenesis. In this study, we aim to investigate the role of TRAF3IP2 in modulating NAMPT expression and explore its downstream impact on promoting cellular energetics in glioblastoma cells. Our results reveal that inhibition of TRAF3IP2 in glioblastoma cells attenuates metabolic activity, as evidenced by decreased expression levels of NAMPT and the mTOR complex, leading to reduction in NAD synthesis and glycolytic function, decreased expression of NAD-dependent deacetylase SIRT1, and increased presence of cellular ROS and expression of tumor suppressor p53, cumulatively resulting in decreased cell viability in glioblastoma. These outcomes elucidate that inhibition of TRAF3IP2 exerts significant anti-tumor effects on glioblastoma by reducing NAD availability and cancer-cell metabolism, highlighting the therapeutic potential of TRAF3IP2 in glioblastoma.

**Graphical Abstract:**

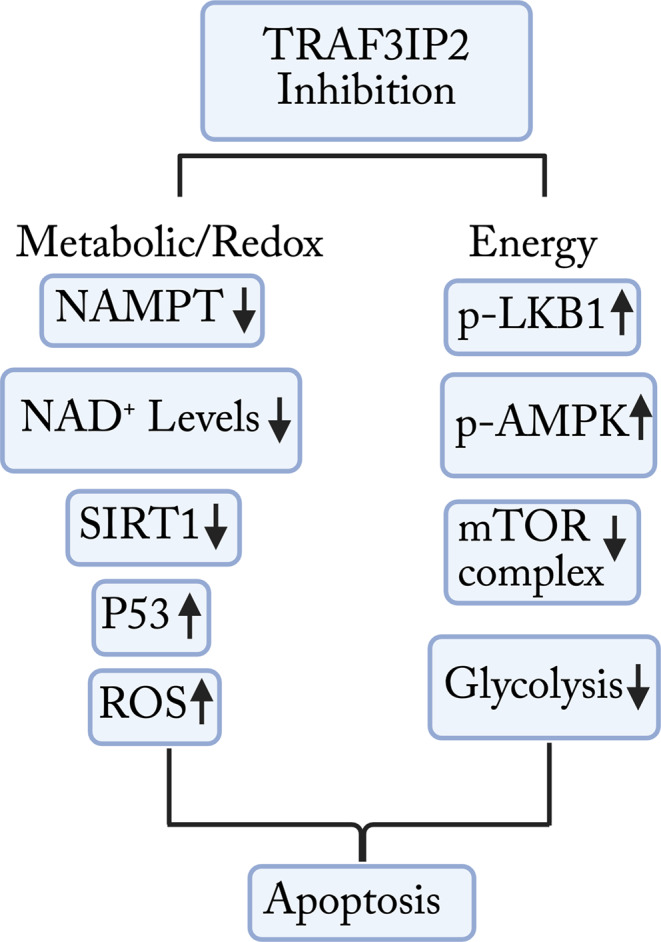

**Supplementary Information:**

The online version contains supplementary material available at 10.1007/s11481-025-10252-z.

## Introduction

Glioblastoma is a grade IV astrocytoma that exhibits an aggressive phenotype, leading to lower disease-free recurrence and overall survival (OS) rates, compared to other brain cancer subtypes (Luckett et al. 2023; Salari et al. 2022). In glioblastoma cells, malignancy is sustained by mutations and defects that causes dysregulated cell cycle progression, resulting in decreased cell cycle arrest and apoptosis, and mitigating harmful levels of reactive oxygen species (ROS) generated from elevated cell replication and protein translation (Hanahan and Weinberg 2000; Rodic and Vincent 2018; Singer et al. 2015). Contrary to non-malignant cells that predominantly rely on mitochondrial respiration for energy, malignant cells, including glioblastoma cells, exhibit increased reliance on glycolysis, a phenomenon known as the Warburg effect (Yaku et al. 2018; Duraj et al. 2021). This process utilizes nicotinamide dinucleotide (NAD) in glycolytic production to generate adenosine triphosphate (ATP) (Stanke et al. 2021; Sharma et al. 2022). The elevated NAD production is facilitated by nicotinamide phosphoribosyltransferase (NAMPT), a key enzyme in the NAD salvage pathway (Chowdhry et al. 2019; Yong et al. 2023; Ye et al. 2020; Sharif et al. 2016). NAMPT catalyzes nicotinamide with 5-phosphoribosyl-1-pyrophosphate, forming nicotinamide mononucleotide (NMN), which is subsequently transformed into NAD, supporting not only glycolysis but also function of NAD-dependent enzymes such as Sirtuin 1 (SIRT1) and DNA repair (i.e. poly(ADP-ribose) polymerase; PARP) (Yaku et al. 2018; Venter et al. 2014). Additionally, NAD contributes to modulating the mammalian target of rapamycin complex (mTORC) signaling pathway, including mTORC1, which is associated with increased protein synthesis (Catena and Fanciulli 2017; Tian et al. 2019). Studies targeting NAMPT in cancer have shown an increase in AMP-activated protein kinase (AMPK) activation, leading to reduction in mTORC1 activity (Garza-Lombó et al. 2018; Indini et al. 2022). This decrease in mTORC1 activity corresponds to reduced protein synthesis and cellular proliferation (Schuster et al. 2015). In addition, studies showed that inhibition of mTORC2 subunit of mTOR (RICTOR) suppress markers of cell metabolism and survival, suggesting crucial role of NAD and its regulatory enzymes in glioblastoma metabolic reprogramming and tumorigenesis (Masui et al. 2013; Sztankovics et al. 2023; Chantaravisoot et al. 2023).

Previously, we reported that the elevated levels of TRAF3IP2, an adaptor protein canonically associated with the IL-17 pathway that acts as a regulator of nuclear factor kappa-light-chain-enhancer of activated B cells (NF-kB), increases expression of inflammatory signaling in glioblastoma cells (Alt et al. 2018). Also, our previous research has shown a strong link between TRAF3IP2 and increased proinflammatory/pro-tumorigenic/proangiogenic signaling in glioblastoma and triple negative breast cancer (TNBC) (Alt et al. 2018; Alt et al. 2020; Izadpanah et al. 2022). While we previously showed that TRAF3IP2 has been implicated in regulating oxidative stress and NAMPT expression in other biological contexts (Das et al. 2018), its direct role in coordinating glioblastoma metabolic reprogramming has not been clarified. In our previous transcriptomic analysis (Alt et al. 2018), TRAF3IP2 knockdown altered expression of multiple genes associated with cellular metabolism, suggesting that TRAF3IP2 regulation extends beyond inflammatory signaling. Importantly, we focused on clinically relevant downstream effectors, NAMPT, NAD, SIRT1, and mTOR, that represent well-established metabolic regulators of glioblastoma growth, survival, and therapy resistance. This approach provides a mechanistic bridge linking TRAF3IP2 activity to central tumorigenic processes without requiring additional exploratory assays on intermediate regulators.

In this study, we examined the impact of targeting TRAF3IP2 on suppression of NAD and NAMPT levels, leading to reductions in glycolysis, ATP production, expression of SIRT1, and mTOR activity. These changes culminated in accumulation of ROS and p53 protein levels that promote cell cycle arrest and apoptosis in glioblastoma. By connecting TRAF3IP2 signaling to core metabolic regulators, our findings provide novel insight into how this adaptor protein drives glioblastoma survival through metabolic reprogramming and highlight its potential as a therapeutic target.

## Methods

### Database Analysis

Gene expression and overall survival was assessed through gene expression data from The Cancer Genome Atlas (TCGA) and Genotype-Tissue Expression (GTeX) and visualized using the Gene Expression Profiling Interactive Analysis (Gepia2; http://gepia2.cancer-pku.cn/) (Tang et al. 2017). Overall survival was analyzed, visualized, and collected from the University of Santa Cruz Xena platform (https://xena.ucsc.edu/) (Goldman et al. 2020).

## Cell Culture & Reagents

Glioblastoma U-87 MG (U87) and U-118 MG (U118) cell lines were purchased from ATCC (Rockville, MD) and Pediatric glioblastoma KNS42 cells (KNS) were obtained from the JCRB (Japan Cancer Research Resources) cell bank. Cell lines were cultured in DMEM (cat# 11965092, Thermo Fisher Scientific, Waltham, MA) supplemented with 10% fetal bovine serum (Peak Serum Inc., Wellington Colorado) and 1% penicillin-streptomycin (cat# 15070063, Thermo Fisher Scientific, Waltham, MA). We selected U87, U118, and KNS cells to represent both laboratory-standard and clinically relevant glioblastoma models. U87 cells, which express high endogenous levels of TRAF3IP2 and NAMPT, facilitate direct comparison with prior metabolic studies. U118 cells harbor p53 mutations, allowing us to evaluate the impact of TRAF3IP2 independent of p53 status. The KNS cell line, characterized by EGFR amplification and CDKN2A deletion, captures the genetic heterogeneity of human glioblastoma. All three lines were employed for the analysis in this study ensuring that targeting TRAF3IP2 yields consistent metabolic and apoptotic responses across diverse glioblastoma backgrounds. Cell lines were transduced with lentiviral vector containing a puromycin resistance sequence and either shRNA TRA3IP2 (TRAF3IP2KD) or scrambled vector control (SCR) as previously described (Alt et al. 2018). Transduced cells were selected using puromycin (cat# AC227420100, Thermo Scientific Chemicals, Waltham MA) and differentiated into the following treatment types: (U87_TRAF3IP2KD_, U87_SCR_; U118_TRAF3IP2KD_, U118_SCR_, KNS _TRAF3IP2KD_, KNS _SCR_). Cells were cultured at 37° Celsius at 5% CO_2_ and the silencing of the TRAF3IP2 was continuously confirmed using qRT PCR and Western blotting on all cell lines. FK866, a noncompetitive NAMPT inhibitor, was purchased from Selleckchem chemical (cat# S2799, Houston, TX).

## Quantitative Real-Time PCR (qRT PCR)

RNA was extracted from cells using RNeasy Micro Kit (cat# 74004, Qiagen, Hilden, Germany). Reverse transcription was synthesized from 1 ug of RNA using iScript cDNA Synthesis Kit (cat# 1708891, Bio-Rad Laboratories, Hercules, CA). Relative mRNA levels were quantified using 10 ng cDNA, iTaq universal SYBR Green Supermix (cat# 1725124, Bio-Rad Laboratories, Hercules, CA) and 100 nM of oligonucleotide primer (Sigma-Aldrich, St. Louis, MO). Fold change was assessed using the ΔΔCT method, with β-actin serving as a housekeeping gene for normalization. The primers used are listed in Table 1.

**Table 1:** Note. This data is mandatory

Primer targets	Forward primer	Reverse primer
NAMPT	5’AGTGGTGCCTGTGTATT-3’	5’TGCCTGTATCTGTGGTCAG-3’
SIRT1	5’AAGGAAAACTACTTCGCAAC-3’	5’GGAACCATGACACTGAATTATC-3’
BIRC5	5’TGTCTCCTCATCCACCTGAA-3’	5’TCCCTGGCTCCTCTACTGTT-3’
mTOR	5’GGAGGAGAAATTTGATCAGG-3’	5’GGGCAACAAATTAAGGATTG-3’
RPTOR	5’CGGAGTTTCCTTTAACAGTG-3’	5’CTGTTGAGTACTTTCATGGC-3’
RICTOR	5’AAATGCATGAAGAAGCAGAG-3’	5’AACAGTGTACAGAAGATACTCC-3’
β-actin	5′CTGGAACGGTGAAGGTGA-3′	5′AAGGGACTTCCTGTAACA-3′

## NAD Quantification

NAD was quantified using an NAD/NADH quantitation kit (cat# MAK037-1KT). Briefly, cells (2 × 10^5^) were harvested from scramble vectors control (SCR) and silenced (TRAF3IP2KD) groups, with both groups treated with either FK866 (100nM) or DMSO vehicle (concentration < 0.001%) (U87_TRAF3IP2KD+DMSO_, U87_SCR+DMSO_; U87_TRAF3IP2KD+FK866_, U87_SCR+FK866_). NAD was isolated and quantified according to colorimetric assay protocol, and the percentage of relative NAD concentration in each group was normalized to the SCR vehicle treated group. Optical density was measured using BMG Labtech’s FLUOstar Optima multi-detection microplate reader (BMG Labtech, Cary, NC).

## Extracellular Flux Assay

Live metabolic activity was assessed using the Agilent Seahorse XFe24 Analyzer (Agilent Technologies, Santa Clara, CA). Glycolysis was assessed with the Seahorse XF Glycolytic Rate Assay Kit Cells (cat# 103344-100), and mitochondrial stress was assessed using the Seahorse XF Mito Stress Test Kit (cat# 103015-100). Cells were seeded overnight prior in a confluent monolayer.

### ROS Detection

Treatment and control cells were seeded overnight in equal quantities in a 96 well plate. ROS production was assessed 30 min post-incubation with 20 μm of 2,7-Dichlorodihydrofluorescein diacetate (DCFH-DA; cat# NC1476516, Cayman Chemical, Ann Arbor, MI), and replaced with DMEM lacking phenol red (cat# 31053028, Thermo Fisher Scientific, Waltham, MA). Plates were then analyzed using BMG Labtech’s FLUOstar Optima multi-detection microplate reader (BMG Labtech, Cary, NC).

## Western Blot

Protein was collected from cell cultures using Mammalian Protein Extraction Reagent (Cat# 78503, Thermo Fisher Scientific, Waltham, MA) and Proteinase Inhibitor Cocktail (Cat# P8340, Sigma-Aldrich, St. Louis, MO). Equal quantities of protein were added to wells in a Mini-Protean TGX Precast Protein Gels (Bio-Rad Laboratories, Hercules, CA) and transferred to PVDF membrane (cat# # 1620177, Bio-Rad Laboratories, Hercules, CA). Membranes were blocked with 5% w/v concentration of either Bovine Serum Albumin (cat# A7906-50G, Thermo Fisher Scientific, Waltham, MA) or nonfat dry milk (cat# 1706404, Bio-Rad Laboratories, Hercules, CA) for one hour, then incubated with primary antibodies overnight at 4 °C. Membranes were washed with TBST (cat# 1706435, Bio-Rad Laboratories, Hercules, CA) with 0.5% added triton-X (cat# T8787, Sigma-Aldrich, St. Louis, MO) and incubated with secondary antibodies for one hour at room temperature. Following another wash with TBST, chemiluminescent signaling was generated using Clarity Western ECL Substrate (cat# 1705060 Bio-Rad Laboratories, Hercules, CA) and imaged using the Chemidoc imaging system from Bio-Rad Laboratories. Relative intensity was assessed using Imagej (Schneider et al. 2012), with antibody intensity normalized to β-actin intensity.

### In Vivo Studies and Immunohistochemistry

U87_TRAF3IP2KD_ and U87_SCR_ cells (1 × 10^6^) were injected in the flank of immunodeficient NSG (NOD scid gamma; The Jackson Laboratory; *n* = 6/group) mice according to the previous reports Following tumor development, the tumor tissues were harvested for histology analysis according to standard protocol. 

Table 2 lists the antibodies used for immunohistochemistry and western blot.

**Table 2:** Note. This data is mandatory

Antibody	Catalog number	Vendor
TRAF3IP2	a6776	Abclonal, Woburn, MA
Sirt1	A17307	Abclonal, Woburn, MA
AMPKa1/AMPKa2	A17290	Abclonal, Woburn, MA
Phospho-AMPK (p-AMPK)	AP0116	Abclonal, Woburn, MA
LKB1	A2122	Abclonal, Woburn, MA
Phospho-LKB1 (Phospho-STK11-S428)	RK05794	Abclonal, Woburn, MA
Survivin	A1551	Abclonal, Woburn, MA
β-actin	AC004	Abclonal, Woburn, MA
p53	MABE339	EMD Millipore, Burlington, MA
Acetyl-p53 (k382)	Ab75754	Abcam, Cambridge, UK
Caspase-3	Ab32351	Abcam, Cambridge, UK
Phospho-p53 (Ser392)	9281	Cell Signaling Technology, Danvers MA
Cleaved caspase-3	5A1E	Cell Signaling Technology, Danvers MA
Raptor	2280	Cell Signaling Technology, Danvers MA
Phospho-Raptor	89,146	Cell Signaling Technology, Danvers MA
Rictor	2214	Cell Signaling Technology, Danvers MA
Phospho-Rictor	3806	Cell Signaling Technology, Danvers MA

## Annexin V/PI Apoptosis Assay

Cells (U87_SCR_, U87_SCR+FK866_, U87_TRAF3IP2KD_, U87_TRAF3IP2^KD+FK866_) were seeded into 6-well plates (3 × 10^5 cells/well) and treated as indicated. After treatment (24 h), cells were harvested, washed with PBS, and stained with Annexin V-FITC and propidium iodide (PI) following the manufacturer’s protocol. Each condition was analyzed in biological triplicate, *p* < 0.05 was considered significant.

### Statistical Analysis

All statistics were performed comparing at least 3 samples (N > = 3). Statistical data is expressed as the mean ± standard deviation. Unpaired Student’s T-test was used to assess statistical significance between treatments. Statistics were measured and figures generated using Graphpad Prism 10 (Graphpad Software, Boston, MA).

## Results

### Upregulation of TRAF3IP2 and NAMPT Inversely Correlated with Decreased Overall Survival in Glioblastoma

**Fig. 1:**
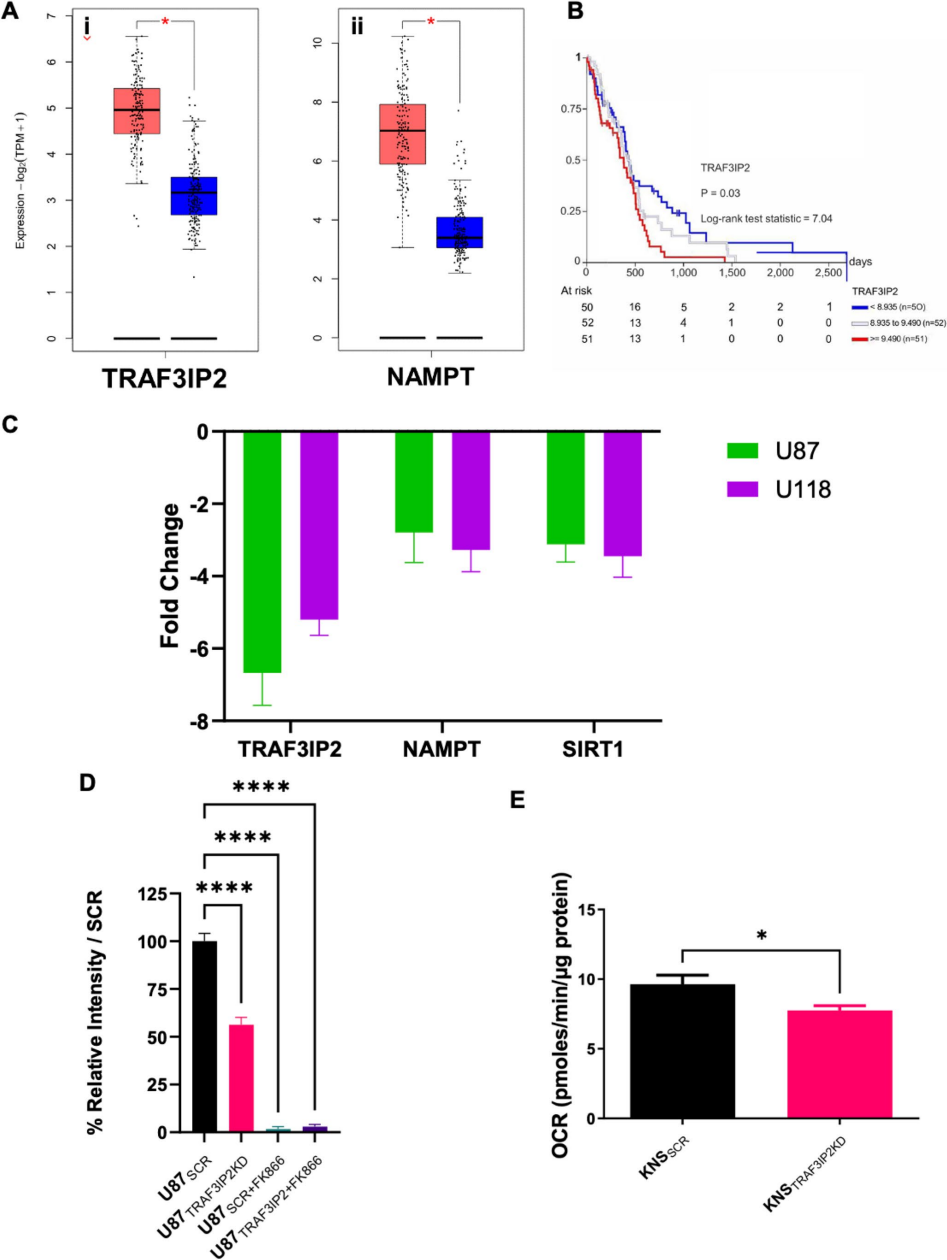
Inhibition of TRAF3IP2 reduces NAMPT expression in glioblastoma. Analysis of glioblastoma samples from TCGA (red bars) compared to non-malignant brain samples (blue bars) from GTEx shows an increase in the expression of TRAF3IP2 and NAMPT mRNA, accessed through GEPIA 2 (http://gepia2.cancer-pku.cn) (**A**). Kaplan-Meier curve analysis of overall survival from TCGA sample analysis using the Xena platform (https://xena.ucsc.edu/) reveals an inverse association between TRAF3IP2 gene expression and length of survival in glioblastoma patients (**B**). Levels of mRNA expression, measured through qRT PCR, show reduced TRAF3IP2, NAMPT and SIRT1 expression in U87_TRAF3IP2KD_ and U118_TRAF3IP2KD_ cells, compared to U87_SCR_ and U118_TRAF3IP2KD_, respectively (*p* < 0.05) (**C**). Silencing TRAF3IP2 reduces relative NAD levels in glioblastoma cell lines, compared to scrambled controls (SCR). The level of NAD was measured in U87_TRAF3IP2KD_ and U87_SCR_ in presence or absence of NAMPT inhibitor “FK866” (DMSO was used as solvent for FK866) (**D**). Silencing TRAF3IP2 decreases ATP production mechanism, measured through reduced oxygen consumption rate (OCR) in Agilent Seahorse XF Cell Mito Stress Test kit, in glioblastoma cell line KNS (Compared to SCR controls) (**E**). Triplicate experiments, *P < 0.05, **P < 0.01, ***P < 0.005, ****P < 0.001

Analysis of the Cancer Genome Atlas (TCGA) and Genotype-Tissue Expression (GTEx) databases demonstrated upregulation of both NAMPT and TRAF3IP2 in glioblastoma compared to normal tissues (Fig. 1A). When stratified by TRAF3IP2 expression, patients with tumors expressing high levels of TRAF3IP2 demonstrated decreased overall survival (OS) (Fig. 1B). *In vitro*, shRNA-mediated knockdown of TRAF3IP2 (TRAF3IP2KD) in U87 and U118 glioblastoma cells led to decreased NAMPT and SIRT1 expression (Fig. 1C), suggesting a correlation between TRAF3IP2 and NAD metabolism via NAMPT.

### Silencing TRAF3IP2 Depletes NAD and Energetic Intermediates in Glioblastoma Cells

To understand the impact of TRAF3IP2 on cellular energetics in glioblastoma, we assessed the metabolomic profile in glioblastoma cells. Through colorimetric analysis, the NAD in U87_TRAF3IP2KD_ cells was quantified and compared to control U87 cells (U87_SCR_). Additionally, we treated U87 cells with NAMPT inhibitor FK866, creating two conditions: U87_SCR_ cells treated with FK866 (U87_SCR+FK866_) and U87_TRAF3IP2KD_ cells treated with FK866 (U87_TRAF3IP2KD+FK866_). Results demonstrate significant decrease in NAD + levels in U87_TRAF3IP2KD_ cells compared to U87_SCR_ cells (Fig. 1D), illustrating the role of TRAF3IP2 in maintaining NAD homeostasis. Furthermore, analysis of mitochondrial stress in KNS cells revealed a reduction in ATP production, indicated by reduced oxygen consumption rate (OCR) following knockdown of TRAF3IP2 (Fig. 1E) suggesting that silencing TRAF3IP2 significantly perturbs cellular energetics in glioblastoma cells.

### Targeting TRAF3IP2 Alters Protein Expression of NAMPT and NAD-Dependent Markers

**Fig. 2:**
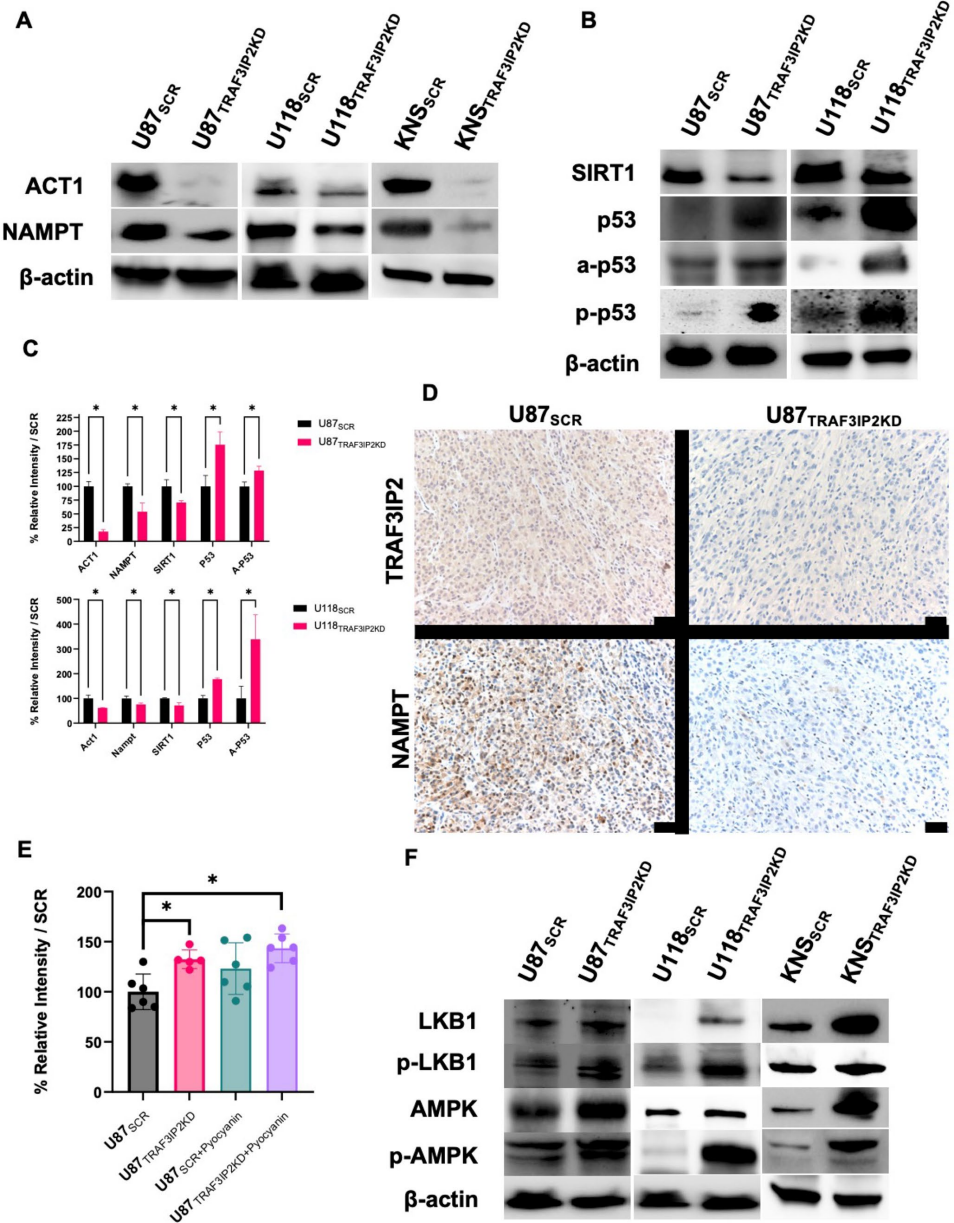
Targeting TRAF3IP2 alters protein levels of NAD salvage pathway markers in glioblastoma. Protein levels of TRAF3IP2 and NAMPT in U87, U118, and KNS cell lines (**A**). Protein levels of SIRT1, total p53, Acetyl-p53[K382] (a-p53), and Phosphorylated-p53[S392] (p-p53) in U87, U118, and KNS cell lines (**B**). Imagej analysis of band intensity in western blots, relative to β-actin (TRAF3IP2KD vs. SCR) (**C**). Immunohistochemical analysis of the tumor tissues demonstrating that U87_TRAF3IP2KD_ -derived tumors had reduced TRAF3IP2 and NAMPT expression, compared to SCR control tumor tissues (Size bar = 50 µM) (**D**). Relative fluorometric intensity of ROS in U87_TRAF3IP2KD_, compared to U87_SCR_ showed that silencing TRA3IP2 increases ROS levels (**E**). Protein expression of LKB1, p-LKB1, AMPK, p-AMPK in transduced U87, U118, and KNS glioblastoma cell lines (F). Triplicate experiments, (*P < 0.05)

**Fig. 4:**
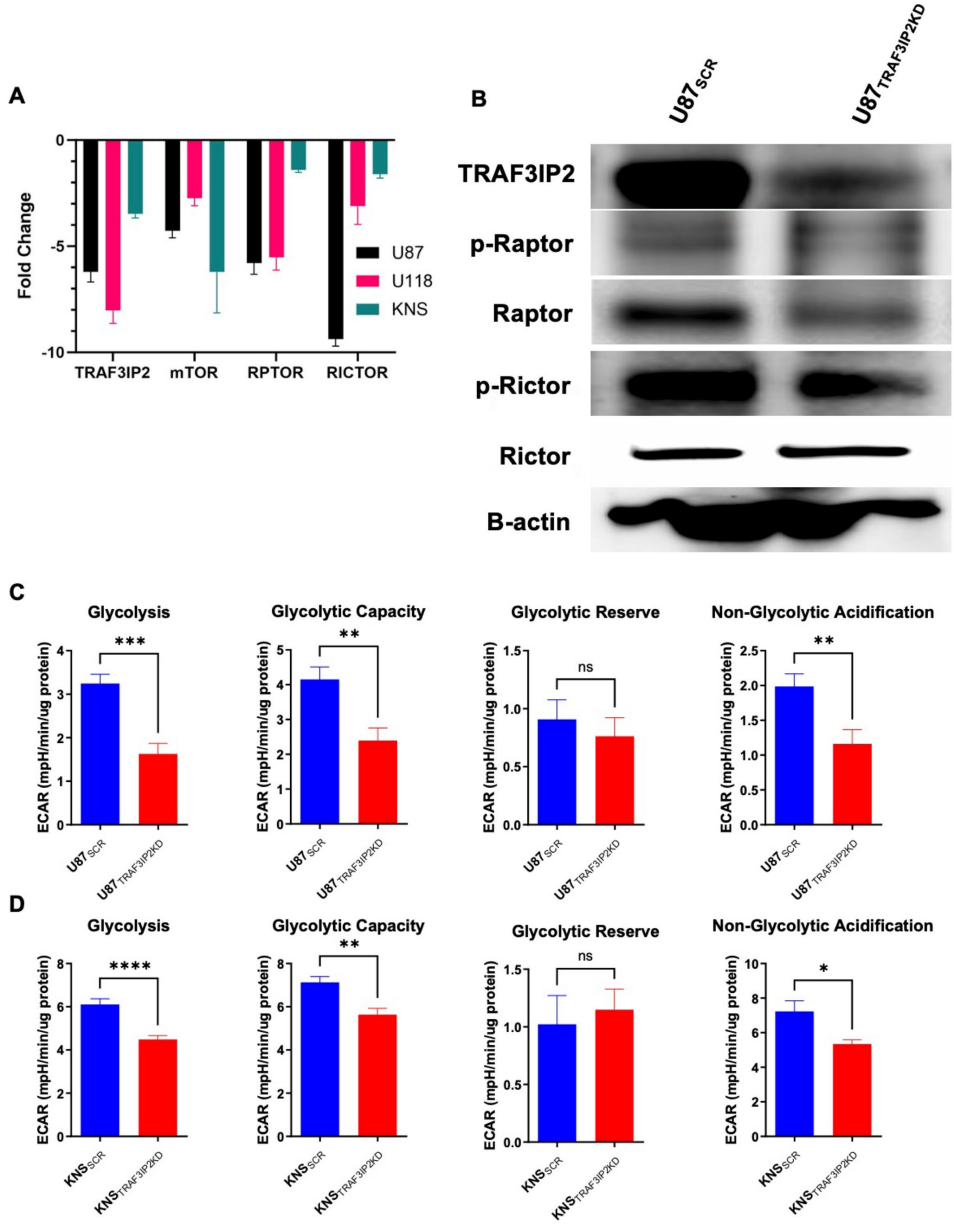
TRAF3IP2 inhibition is associated with increased ROS presence and apoptosis. Gene expression analysis showed elevated levels of survivin in TRAF3IP2KD cells, compared to SCR controls (Triplicate experiments/cell lines, *p* < 0.05) (**A**). Western blot analysis of showed levels of survivin, total caspase-3 and cleaved caspase-3 protein in U87 and U118 cells (**B**). Scatterplot of glioblastoma cell groups and treatment types (U87_SCR+DMSO_ (i); U87_SCR+FK866_ (ii); U87_TRAF3IP2KD+DMSO_ (iii); U87_TRAF3IP2KD+FK866_ (iv)), with bar graph representation of Annexin V and PI staining of U87 (v) (**C**). *P < 0.05

Western blot analysis was conducted to elucidate the effect of targeting TRAF3IP2 on key metabolic and apoptotic regulators in glioblastoma cells. Silencing TRAF3IP2 significantly reduced the expression of NAMPT and SIRT1, while simultaneously increasing total, phosphorylated, and acetylated p53 compared with SCR control cells (Fig. 2A-C). These results indicate a direct correlation between TRAF3IP2 activity, and the expression levels of key markers involved in glioblastoma cellular metabolism and tumor suppression. The decrease in NAMPT expression following TRAF3IP2 knockdown leads to a subsequent reduction in SIRT1 levels, a NAD-dependent deacetylase involved in cellular stress responses and longevity. The reduction in SIRT1 correlates with an increase in both total, phosphorylated, and acetylated forms of p53 resulting in enhanced apoptosis. The increase in acetylated-p53 suggests enhanced activation and stability of p53, contributing to potential anti-tumor effects in glioblastoma cells. Furthermore, cleaved caspase-3 was markedly elevated in TRAF3IP2KD cells, confirming activation of the intrinsic apoptotic pathway, and Annexin V staining (Fig. 4) demonstrated a significant rise in early apoptotic populations, providing complementary evidence of apoptosis.

To elucidate the in vivo implications of targeting TRAF3IP2 on NAMPT expression, we induced tumors using U87_TRAF3IP2KD_ and U87_SCR_ cells. As previously reported, compared to U87_SCR_, U87_TRAF3IP2KD_ induced significantly smaller tumors Immunohistochemistry of resulting tumors showed a notable decrease in TRAF3IP2 and NAMPT levels in tumors derived from U87_TRAF3IP2KD_ cells, in comparison to tumors of U87_SCR_ cells (Fig. 2D). These findings support the inverse relationship between TRAF3IP2 and NAMPT expressions in vivo.

Given the importance of reactive oxygen species (ROS) in glioblastoma biology, we measured intracellular ROS levels using fluorometric assay. Our results show that targeting TRAF3IP2 led to further elevation of ROS in U87_TRAF3IP2KD_ compared to SCR control cells, potentially overcoming the elevated ROS resistance in glioblastoma cells (Fig. 2E). This suggests that TRAF3IP2 plays a critical role in regulating oxidative stress and maintaining redox balance in glioblastoma cells.

### TRAF3IP2 Inhibition Increases AMPK-Associated Expression

To assess whether decline in cellular energetics from silencing TRAF3IP2 in glioblastoma cells affects nutrient-sensing pathways, LKB1 and AMPK protein expression were examined. Results show that silencing TRAF3IP2 in glioblastoma cells increased expression and phosphorylation in LKB1 and AMPK (Fig. 2F). These results indicate that targeting TRAF3IP2 disrupts signaling pathways that are crucial for glioblastoma growth and proliferation. Specifically, TRAF3IP2KD cells, when compared to control cells, demonstrated enhanced expression of phosphorylated LKB1 and AMPK, which are pivotal components of the nutrient-sensing signaling pathway. These results suggest that inhibiting TRAF3IP2 in glioblastoma cells is associated with disruption of metabolomic signaling pathways, thereby potentially impairing tumor growth and proliferation mechanisms that are dependent on these signaling networks.

### Silencing TRAF3IP2 is Associated with a Decrease in mTORC Activity

**Fig. 3:**
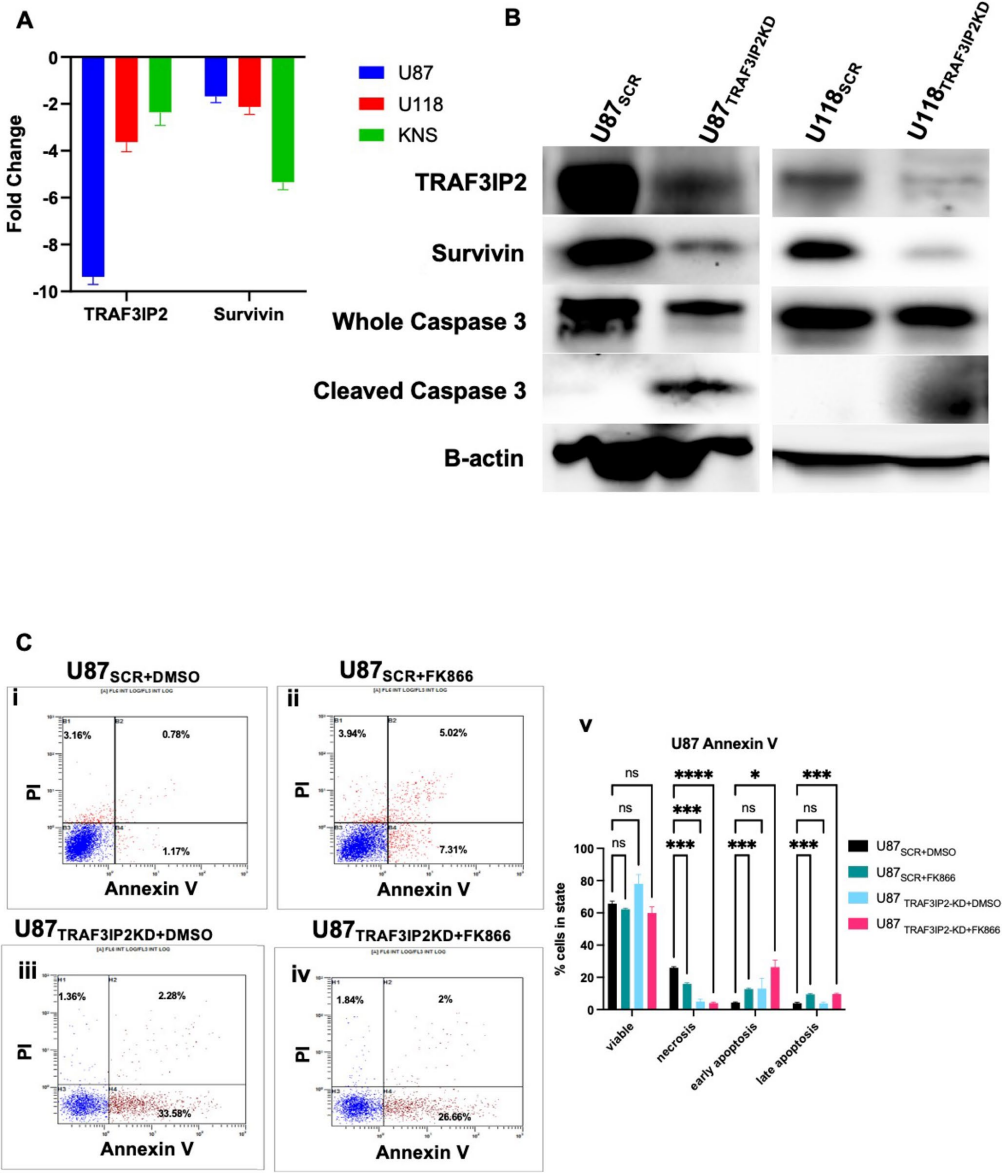
Targeting TRAF3IP2 reduces mTOR associated cell growth markers and glycolysis and glioblastoma cells. Fold change difference in mTOR and mTORC associated markers Raptor and Rictor in TRAF3IP2KD cells, compared to SCR controls, in U87, U118 and KNS cell lines (Triplicate experiments/cell lines, *p* < 0.05) (**A**). Western blot of Raptor, p-Raptor, Rictor, and p-Rictor protein expression in U87_SCR_ and U87_TRAF3IP2KD_ cells (**B**). Agilent Seahorse XF Glycolytic stress test measuring glycolysis, glycolytic capacity, glycolytic reserve, and non-glycolytic acidification through assessment of real-time ECAR levels, normalized to total µg protein in cell culture wells, of glioblastoma cell lines U87 (**C**) and KNS (**D**)

The increased activity of mTOR is a characteristic feature of glioblastoma cells, contributing significantly to their proliferation and survival. We next studied the effects of targeting TRAF3IP2 on mTOR activity. The silencing TRAF3IP2 in glioblastoma cells led to significant decreases in RNA expression of mTOR complex such as Raptor (mTORC1) and Rictor (mTORC2) (Fig. 3A). In addition, western blot analysis shows a decreased expression of phosphorylated both Raptor and Rictor proteins (Fig. 3B). These results show that targeting TRAF3IP2 in glioblastoma is associated with a reduction in mTOR complex expression.

### Inhibition of TRAF3IP2 Reduces Glycolysis in Glioblastoma Cells

Glioblastoma cells predominantly rely on glycolysis for ATP production, a metabolic pathway that supports glioblastoma cell rapid proliferation and survival under hypoxic conditions typical to tumor microenvironment. Analysis of glycolytic stress in glioblastoma cell lines (U87_TRAF3IP2KD_ vs. U87_SCR_, and KNS_TRAF3IP2KD_ vs. KNS_SCR_,) shows a significant decline in both glycolysis and glycolytic capacity upon TRAF3IP2 knockdown (Fig. 3C-D). This result indicates the efficacy of targeting TRAF3IP2 in diminishing glycolytic function in glioblastoma cells.

### Targeting TRAF3IP2 Progresses Apoptotic Signaling and Leads Toward Cell Death

Further analysis underscores the effects of targeting TRAF3IP2 in suppression of survivin, a marker of cell proliferation, in glioblastoma cells (Fig. 4A). The reduction of surviving in RNA levels was also shown at the protein level, with western blot analysis displaying a significant decrease in survivin protein expression in U87_TRAF3IP2KD_ and U118_TRAF3IP2KD_ cells (Fig. 4B). Moreover, an increase in cleaved caspase-3 was observed, indicating an upregulation of apoptotic activity (Fig. 4B). These results collectively show that inhibiting TRAF3IP2 directly impacts survivin expression, leading to initiation of apoptotic activity through increased cleaved caspase-3.

Further exploration into the consequences of altering NAD metabolism through NAMPT inhibition revealed its impact on cell viability. In addition, Annexin V/PI assays post-FK866 treatment showed a significant elevation in both early and late apoptotic populations in TRAF3IP2KD cells (Fig. 4C), further corroborating enhanced apoptotic signaling. These results indicate that silencing TRAF3IP2 not only disrupts NAD metabolism but also potentiate apoptotic progression beyond the effects of NAMPT inhibition alone.

## Discussion

The novel data from this study shows the effect of TRAF3IP2 in regulation of metabolic function in glioblastoma. Previously, we showed the increased TRAF3IP2 expression levels in glioblastoma relative to non-malignant cells and demonstrated that inhibition of TRAF3IP2 restricts tumor growth (Alt et al. 2020; Izadpanah et al. 2022). Results from this study suggest TRAF3IP2 as a pivotal regulator of cellular metabolism. Analysis of TCGA and GTEx databases demonstrated a significant upregulation of TRAF3IP2 and NAMPT in glioblastoma tissues, inversely correlated with overall survival (Fig. 1A-B). This association suggests that TRAF3IP2 not only plays a role in tumor growth and survival but may also serve as a prognostic marker for glioblastoma (Hanahan and Weinberg 2000; Alt et al. 2018, 2020; Izadpanah et al. 2022). Our data indicate that inhibition of TRAF3IP2 is associated with cellular metabolism through reduced NAMPT expression in glioblastoma cells (Fig. 1C), leading to a downstream reduction of NAD (Fig. 1D) and ATP levels (Fig. 1E). This reduced metabolic function leads to decreased glycolytic function in glioblastoma cells (Fig. 3C-D). The direct relationship between TRAF3IP2 knockdown and reduced NAMPT expression underscores a novel regulatory axis critical for maintaining NAD homeostasis and supporting the energetic demands of glioblastoma cells. While TRAF3IP2 has been implicated in oxidative stress and metabolic regulation in other disease contexts (e.g., cardiovascular stress) (Das et al. 2018), its role in glioblastoma metabolism has not been fully elucidated. Prior work has proposed TRAF3IP2 as a therapeutic target in glioma, primarily linking it to inflammatory and proliferative signaling. Our findings extend these observations by demonstrating that downstream metabolic effectors, including NAMPT, NAD, SIRT1, and mTOR, are directly regulated by TRAF3IP2 in GBM cells. These molecules were chosen because they are well-established drivers of glioblastoma survival, metabolic fitness, and therapy resistance, and their coordinated regulation provides a mechanistic bridge connecting TRAF3IP2 to core tumorigenic processes without requiring interrogation of every intermediate step. Thus, by focusing on clinically relevant downstream readouts, our work highlights the biological significance of TRAF3IP2 in glioblastoma metabolism and its translational potential.

These observations are consistent with previous studies highlighting the role of NAMPT in cancer metabolism and its potential as a therapeutic target in various malignancies (Schuster et al. 2015; Barraud et al. 2016; Sawicka-Gutaj et al. 2015; Guo et al. 2019; Wei et al. 2022). The reduction in ATP production and NAD levels following inhibition of TRAF3IP2 indicates a disruption of metabolic processes crucial for tumor cell survival (Amin et al. 2021; Udawant et al. 2021). This is further supported by our observations of decreased glycolytic function (Fig. 3C-D) and mTORC activity (Fig. 3A-B), both of which are vital for the proliferation and maintenance of cancer cells under hypoxic conditions typical of the glioblastoma microenvironment(Emami Nejad et al. 2021; Monteiro et al. 2017). These results align with the emerging consensus that targeting metabolic reprogramming in cancer cells can limit their survival and proliferation (Marallano et al. 2024; Sanzey et al. 2015).

Furthermore, decreased NAD production following targeting TRAF3IP2 also leads to inhibition of SIRT1 protein expression while increasing p53 expression (Fig. 2B), which results in decreased ROS clearance (Fig. 2E) and cell viability, leading to increased apoptosis observed in cells inhibited with TRAF3IP2 (Fig. 4C). The decrease in NAMPT and SIRT1 levels, coupled with increased p53 acetylation upon TRAF3IP2 knockdown, suggests a shift towards tumor suppression (Chen et al. 2019; Ma et al. 2017). Furthermore, it has been shown that inhibition of NAMPT and SIRT1 leads to ROS accumulation, progressing glioblastoma cells toward apoptosis and sensitizing cells to chemotherapy (Chen et al. 2019; Wang et al. 2020; Dong et al. 2008). The observed increase in acetylated p53, indicative of enhanced activation and stability of p53 (Li et al. 2024), points to a potential mechanism by which TRAF3IP2 inhibition could exert anti-tumor effects (Fig. 5A). The synergistic effects observed with TRAF3IP2 knockdown and NAMPT inhibition on reducing cell viability and increasing apoptosis suggest a novel combination strategy that warrants further exploration. Additionally, the impact of TRAF3IP2 inhibition on increased phosphorylation of AMPK, LKB1, and oxidative stress presents opportunities to disrupt glioblastoma cell adaptation to metabolic and environmental challenges and reduce cell proliferation (Shoda et al. 2023; Zhang et al. 2010; Guo et al. 2009; Liu et al. 2022). The inclusion of cleaved caspase-3 and Annexin V data further validates that TRAF3IP2 knockdown robustly triggers apoptotic pathways beyond p53 activation. Taken together, these findings provide new evidence that TRAF3IP2 serves as a master regulator connecting inflammatory signaling with metabolic reprogramming in glioblastoma. This work therefore fills a critical gap in the existing literature by demonstrating that TRAF3IP2-driven regulation of NAMPT and NAD metabolism is not just correlative but mechanistically tied to tumor viability. Future studies should focus on validating these findings in vivo and exploring the clinical applicability of TRAF3IP2 targeting in glioblastoma. Investigating the interactions between TRAF3IP2 and other metabolic regulators could also unveil new targets within the complex network of cancer metabolism.

## Conclusion

The results of this study demonstrate the mechanistic role of TRAF3IP2 on regulation of metabolism in glioblastoma cells. We showed that targeting TRAF3IP2 in glioblastoma inhibits NAMPT expression, reducing NAD levels, glycolytic function, NAD-dependent SIRT1 expression, and mTORC-associated pathways promoting growth, leading to increased p53 expression, ROS presence, and progression toward cell death. Moreover, the combined evaluation of p53 activation, cleaved caspase-3 induction, and Annexin V positivity confirms that TRAF3IP2 knockdown elicits a multifaceted apoptotic response, reinforcing its promise as a target for future therapeutic development. Cumulative with our other previous reports, these data indicate that TRAF3IP2 may play important role in multiple tumorigenic pathways, suggesting TRAF3IP2 as a potential therapeutic target for glioblastoma.

### Limitations

While this study establishes TRAF3IP2’s role in regulating metabolism and apoptosis in established glioblastoma cell lines, several limitations warrant mention: our conclusions rely primarily on in vitro assays and short-term xenograft models, necessitating long-term patient-derived xenograft studies and metabolic-flux analyses to confirm translational relevance; rescue experiments with NAD precursors (e.g., NMN or nicotinamide) to definitively prove pathway specificity have not yet been performed and are planned in ongoing work; and although we assessed multiple GBM lines with distinct genetic backgrounds, validation in primary tumor specimens and across additional molecular subtypes is needed to ensure the broad applicability of our findings. Addressing these points in future studies will be essential for advancing TRAF3IP2 inhibition toward a viable therapeutic strategy in glioblastoma.

## Supplementary Information

Supplementary Material 1.

## Data Availability

No datasets other than those presented in this manuscript were generated or analysed during the current study.

## Abbreviations

TRAF3IP2: TRAF3 interacting protein 2
NF-kB: Nuclear factor kappa-light-chain-enhancer of activated B cells
NAMPT: Nicotinamide phosphoribosyltransferase
SIRT1: Sirtuin 1
p53: Tumor protein p53
a-p53: Acetylated P53 (K382)
p-p53: Phosphorylated p53 (S392)
LKB1: Liver Kinase B1
p-LKB1: Phosphorylated Liver Kinase B1
AMPK: AMP-activated protein kinase
p-AMPK: Phosphorylated AMP-activated protein kinase
mTOR: Mammalian target of rapamycin
mTORC: Mammalian target of rapamycin complex
RPTOR: Raptor gene
β-actin: Actin beta
ROS: Reactive oxygen species
NAD: Nicotinamide adenine dinucleotide
NMN: Nicotinamide mononucleotide
ADP: Adenosine diphosphate
ATP: Adenosine triphosphate
NMN: Nicotinamide mononucleotide
OCR: Oxygen consumption rate
U87: Glioblastoma cell line
U118: Glioblastoma cell line
KNS: Glioblastoma cell line
TCGA: The Cancer Genome Atlas
GTeX: Genotype-Tissue Expression
NSG: NOD scid gamma
DMSO: Dimethyl sulfoxide
DCFH-DA: Dichlorodihydrofluorescein diacetate
PI: Propridium iodide
FK866: NAMPT inhibiting molecule
