# Supplementary figures and images for: Inhibition of TRAF3IP2 Modulates NAMPT and NAD Metabolism in Glioblastoma
Source: J Neuroimmune Pharmacol. 2025 Oct 22;20(1):95. doi: 10.1007/s11481-025-10252-z (PMC12546416; doi:10.1007/s11481-025-10252-z)

## Supplementary Figures

Figure 2A

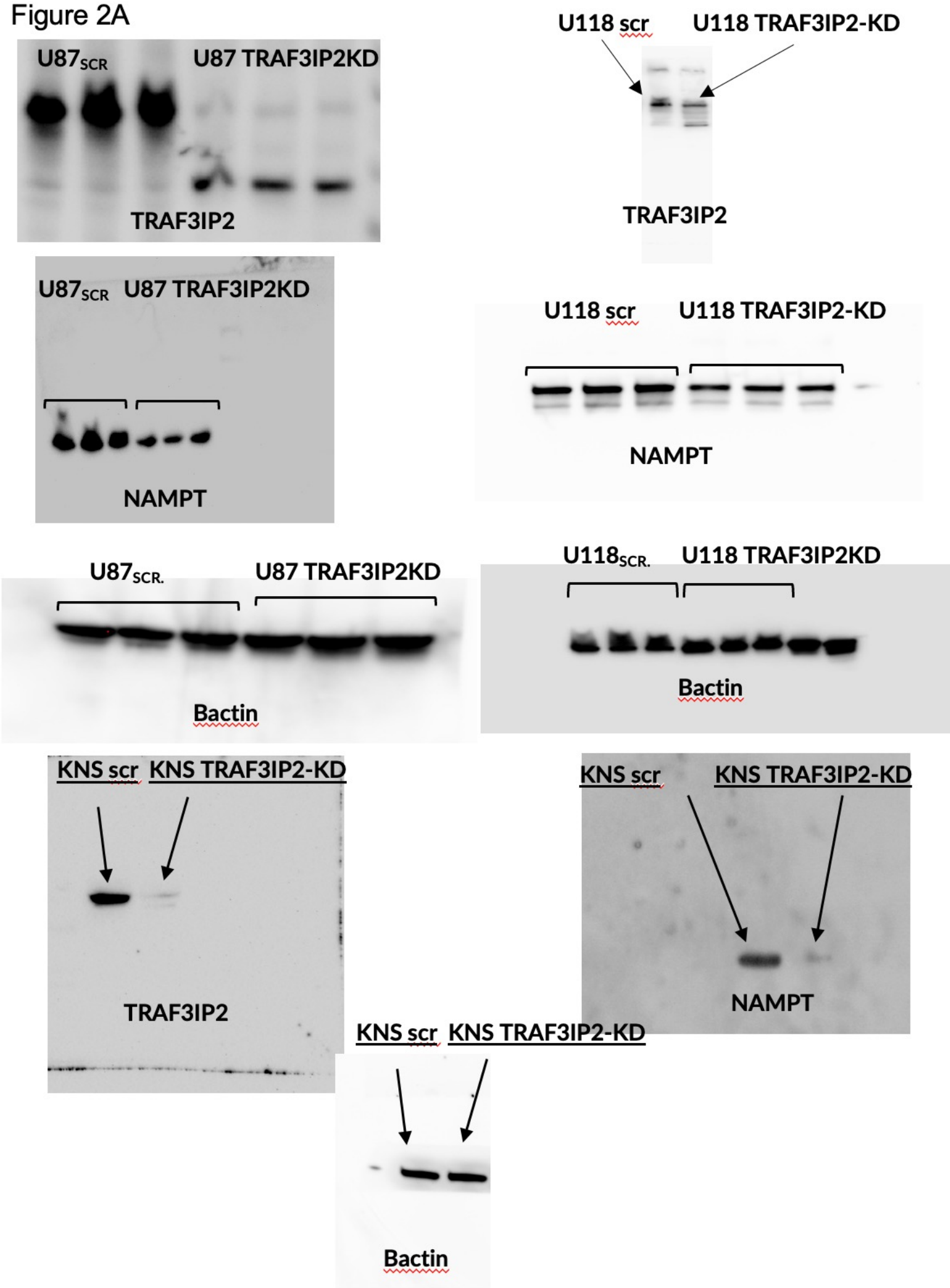

Figure 2B

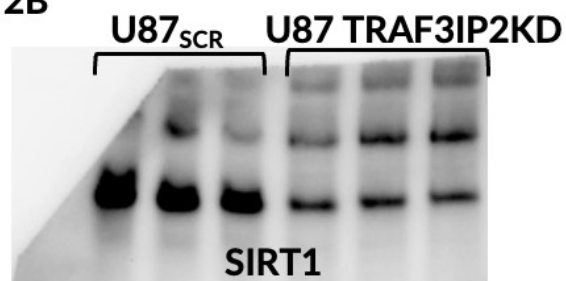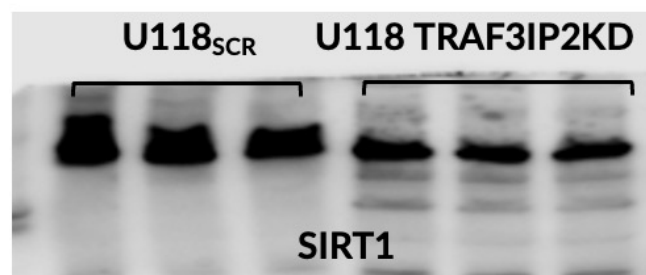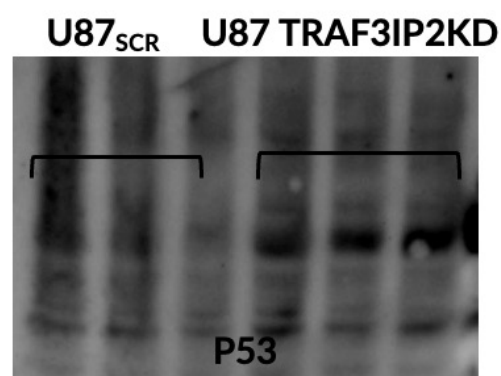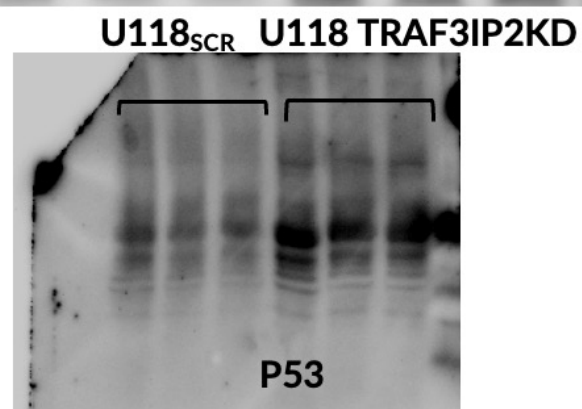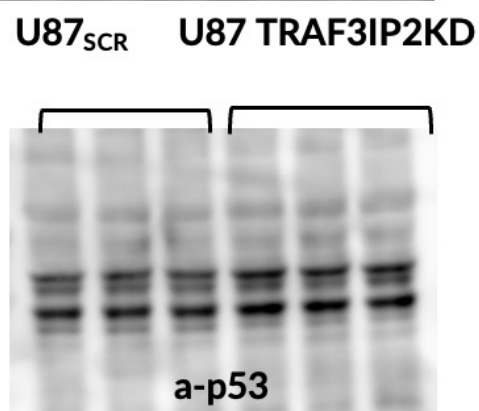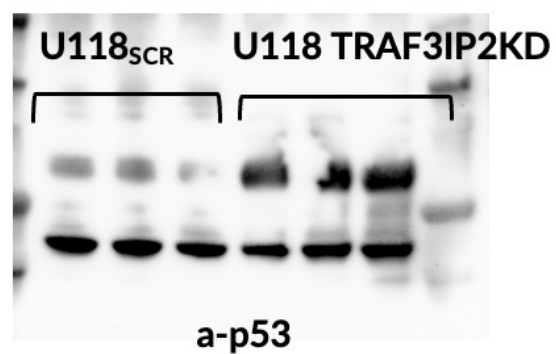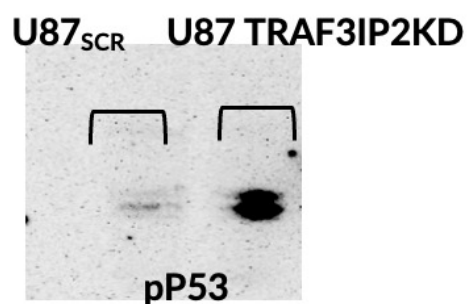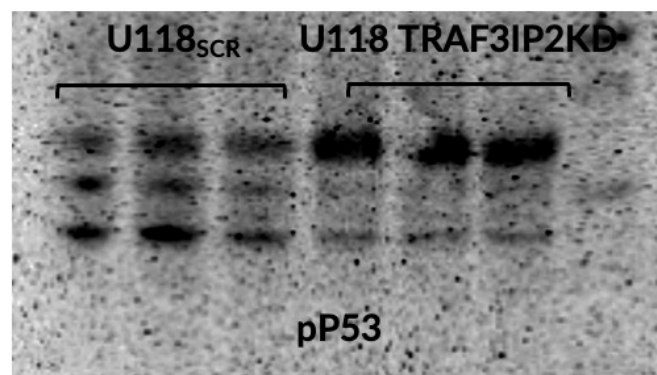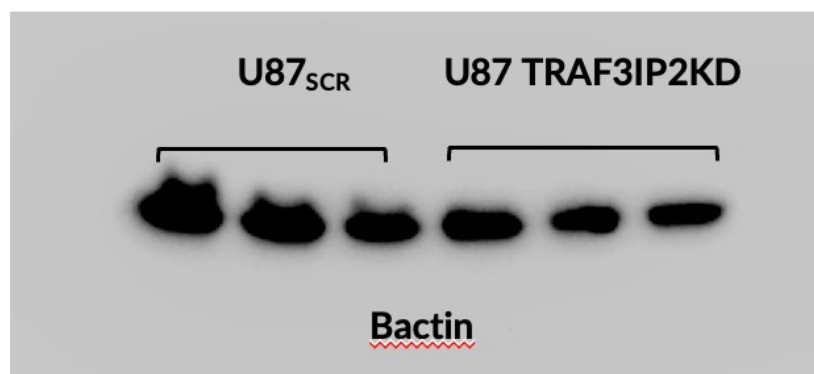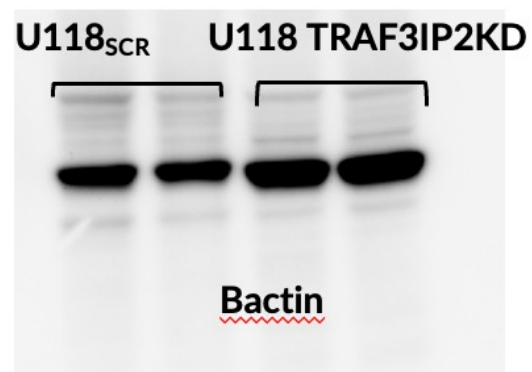

Figure 2F

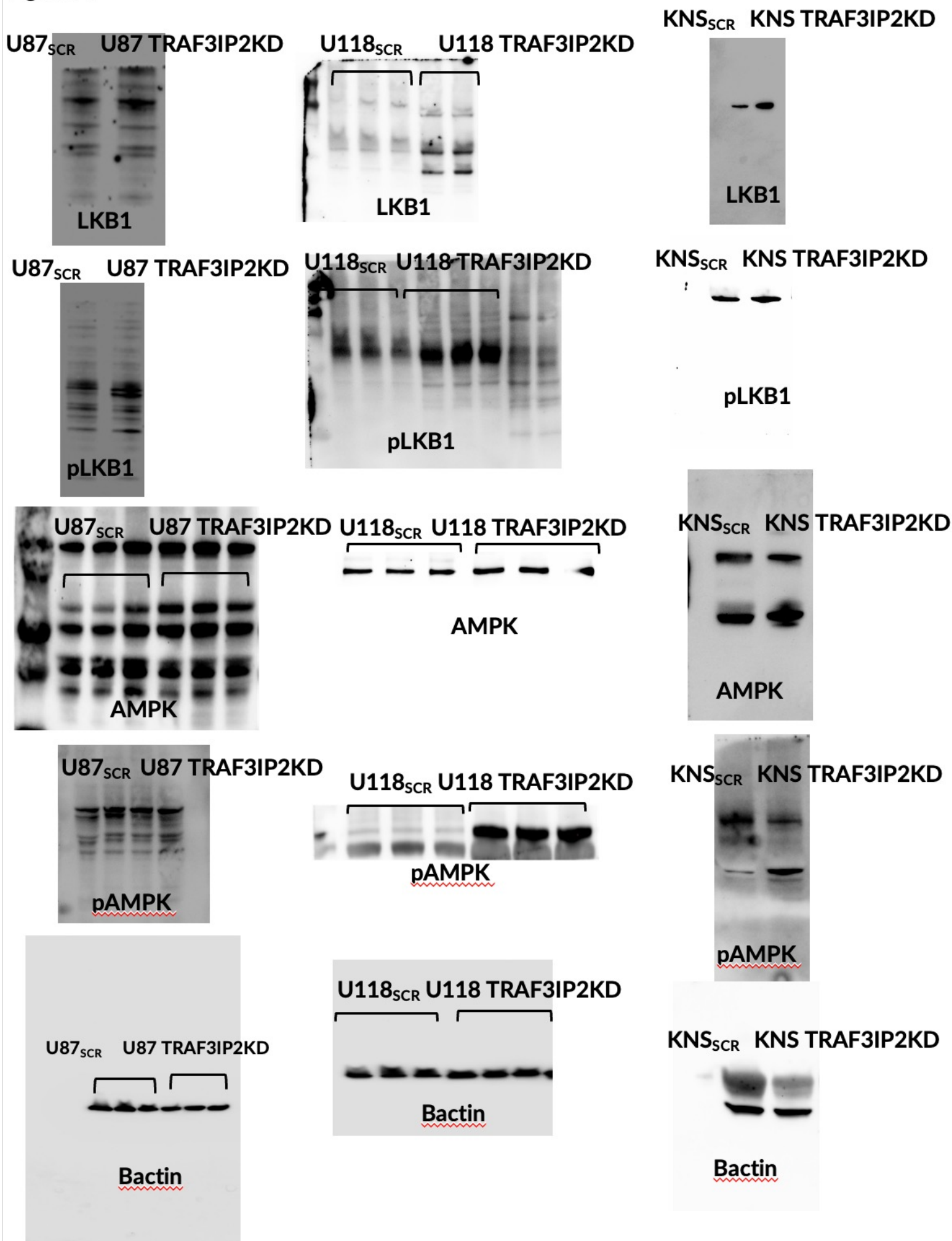

Figure 3B

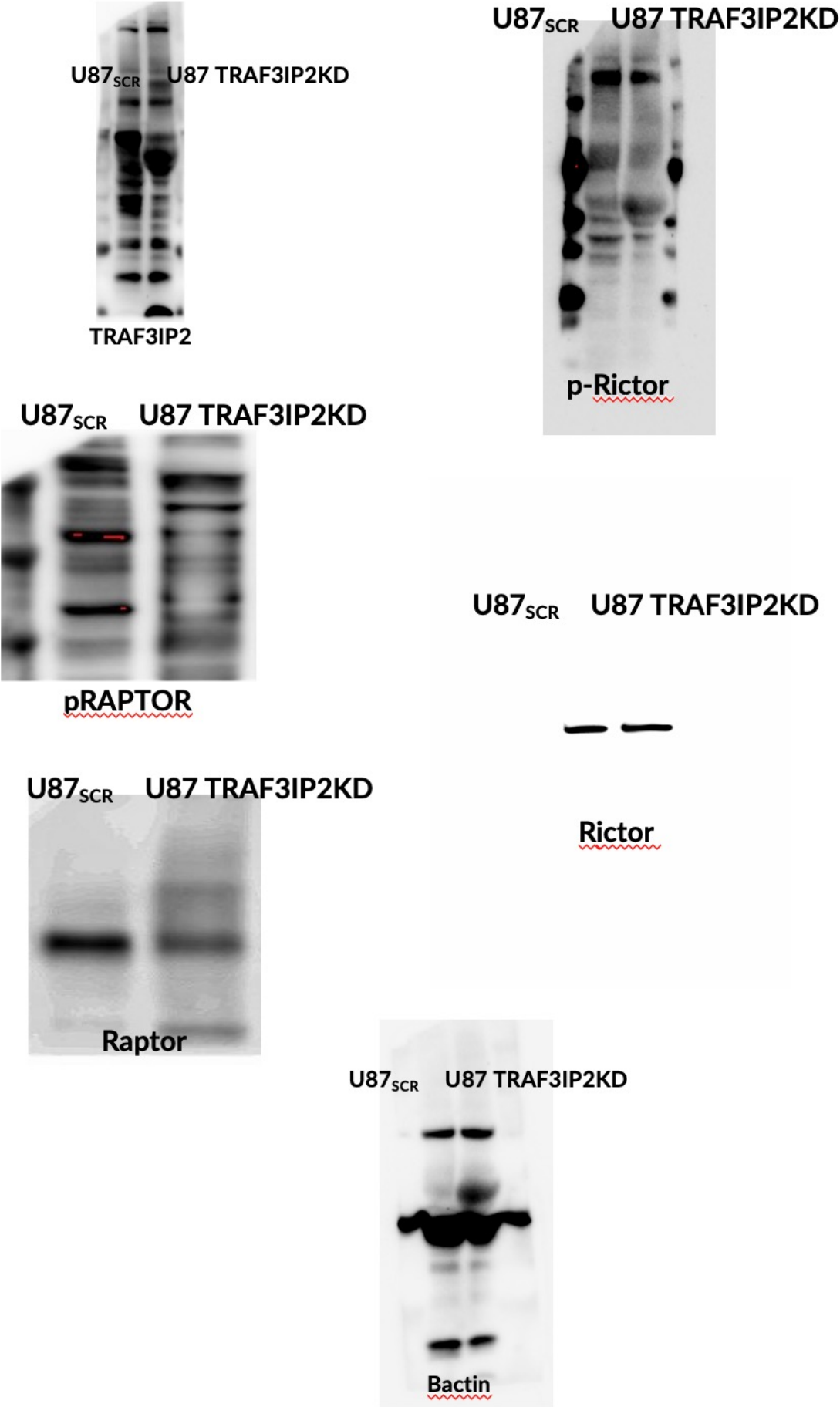

Figure 4B

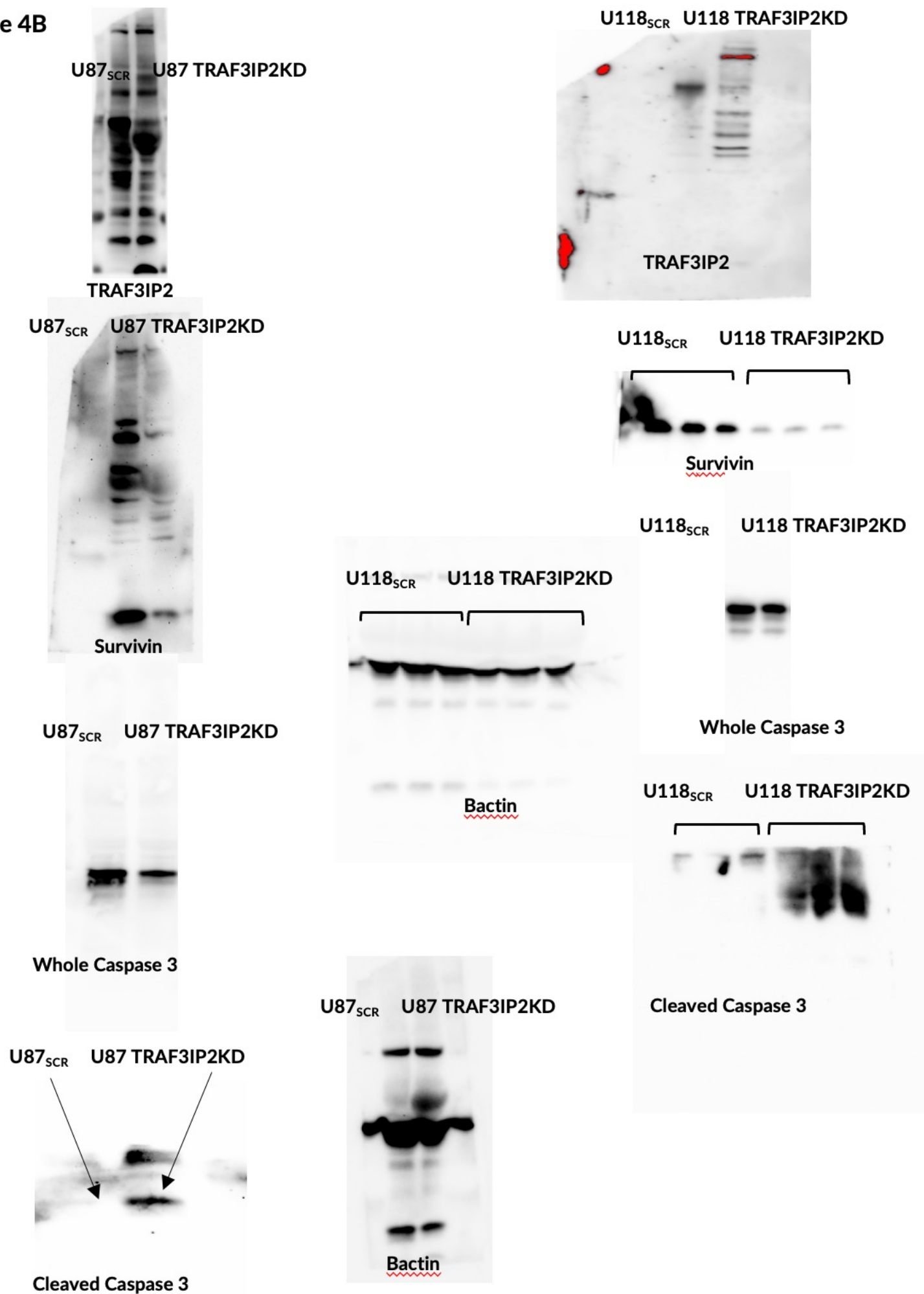

Supplement: Supplementary file 1 — Supplementary Material 1. [file 11481_2025_10252_MOESM1_ESM.pdf]
